# Comparison of characteristics and tumor targeting properties of extracellular vesicles derived from primary NK cells or NK-cell lines stimulated with IL-15 or IL-12/15/18

**DOI:** 10.1007/s00262-022-03161-0

**Published:** 2022-02-04

**Authors:** Miriam Aarsund, Filip M. Segers, Yunjie Wu, Marit Inngjerdingen

**Affiliations:** 1 Department of Pharmacology, Institute of Clinical Medicine, University of Oslo, Oslo, Norway; 2 Department of Pharmacology, Clinic of Laboratory Medicine, Oslo University Hospital, Oslo, Norway

**Keywords:** NK cells, EVs, NKG2D, IL-15, IL-12, IL-18

## Abstract

NK cell-based therapies have shown promise for hematological cancer forms, but their use against solid tumors is hampered by their poor ability to infiltrate the tumor. NK cells release extracellular vesicles (EVs) containing cytolytic proteins, indicating that NK-cell derived EVs may have therapeutic potential. In this study, we compared the tumor-targeting potential of EVs derived from either primary NK cells or the NK cell lines NK-92 and KHYG-1 cultured in IL-15 alone or in combination with IL-12 and IL-18. Primary NK cells were also stimulated through the activating receptor CD16. Tumor cell apoptosis was measured using a panel of human colon, melanoma, glioblastoma, prostate, breast, and ovarian tumor cell line spheroids. NK cells or NK-92 cells stimulated with IL-12, IL-15, and IL-18 generated EVs with higher efficiency than EVs from resting cells, although similar amounts of EVs were produced under both conditions. Proteomic analysis indicated similar distribution of cytolytic proteins in EVs from primary NK cells and NK-92, but lower levels in KHYG-1 EVs that translated into poor capacity for KHYG-1 EVs at targeting tumor cell lines. Further, we show that CD16-stimulated NK cells release low amounts of EVs devoid of cytolytic proteins. Importantly, EVs from cytokine-stimulated NK cells penetrate into the spheroid core, and tumor spheroid susceptibility to NK-cell derived EVs was linked to differential expression of the NKG2D ligands MICA/B, which was blocked with an anti-NKG2D antibody. We conclude that EVs from activated primary NK cells or NK-92 cells has the best potential to infiltrate and target solid tumors.

**Supplementary Information:**

The online version contains supplementary material available at 10.1007/s00262-022-03161-0.

## Introduction

NK cells are innate lymphocytes with cytolytic activity against virally infected or malignant cells. Their activity is controlled by activating and inhibitory receptors [1]. NK cells target stress-induced ligands expressed by transformed cells via a diverse repertoire of activating receptors such as NKG2D, NKp30, NKp46, and DNAM-1. Target cell death is induced by cytolytic proteins secreted from cytolytic granules from within NK cells, or directly by death receptor-induced apoptosis [2]. NK cell activity is further modulated by cytokines. The cytokine IL-15 is necessary for NK cell survival and proliferation, and combining IL-15 with IL-12 and IL-18 induces potent IFN-γ production [3]. Short-term IL-12/15/18-stimulated NK cells differentiate into memory-like NK cells with heightened ability for cytokine secretion [4], and also enhanced capacity to target cancer cells as shown in both pre-clinical and clinical studies [5–8]. NK cells are now exploited clinically in several immunotherapeutic strategies for cancer, with particular promising results for myeloid malignancies [9, 10]. Adoptive NK cell transfer to patients with solid tumors have shown less success, despite a number of clinical trials [11–13]. The microenvironment of solid tumors poses a challenge to NK cell-based therapies due to suppressive signals, resulting in poor infiltration of NK cells, and suppression of NK cell effector functions [14]. Based on these challenges, strategies exploiting NK-cell derived extracellular vesicles (EVs) could represent a promising approach.

EVs have gained increasing attention due to their unique role in intercellular communication [15–17]. EVs are membrane bound vesicles carrying proteins, lipids, small nuclei acids, and metabolites from the secreting cell, and originate from either inside the cell in the form of exosomes or bud off from the plasma membrane as microvesicles [18, 19]. A variety of therapeutic applications of EVs have been reported [20]. With regard to anti-tumor strategies, EVs isolated from immune cells may represent promising immunotherapeutic agents. Exosomes from dendritic cells have been exploited in vaccine approaches, and shown therapeutic potential in patients suffering from metastatic melanoma and non-small cell lung cancer [21, 22]. Macrophage-derived EVs loaded with cytostatics have been demonstrated to target pulmonary metastases in mice models [23].

NK-cell derived EVs are demonstrated to induce apoptosis of cancer cell lines. These studies have exploited either primary NK cells or the FDA-approved NK-92 cell line as source for generating NK-EVs. The cells have typically been expanded with IL-2 and/or IL-15, and in some instances with feeder cells [24–30]. NK-EVs variably kill cancer cell lines of both hematological and solid tumor origin [17, 24]. Moreover, two independent studies showed tumor regression of neuroblastoma in mice treated with NK-EVs [28, 31]. A series of studies suggest that NK-cell derived EVs induce tumor-dell death via their incorporation of cytotoxic proteins [17, 25, 26, 28]. Thus, NK-EVs are equipped to induce apoptosis in target cells, but the specific mechanisms for how NK-EVs interact with and kill the cancer cell remains unclear.

As IL-12/15/18-activated NK cells have shown potent anti-tumor activities against a wide range of tumors, this motivated us to compare the effect of EVs derived from resting (cultured in IL-15 alone) versus IL-12/15/18-activated NK cells. Moreover, there has been little standardization of NK-EV products, making it difficult to compare different studies on NK-EVs. In this study, we provide a comparative evaluation of the characteristics and functional activity of EVs separated from primary NK cells and the NK cell lines NK-92 and KHYG-1 stimulated with either IL-15 or IL-12/15/18 in order to identify the most potent EV product.

## Materials and methods

### Primary cells and cell lines

Buffy coats were obtained from healthy human donors from the blood bank at Oslo University Hospital according to the Declaration of Helsinki. The study was approved by the South-Eastern Norway Regional Ethical Committee (REK2012-1452). Primary NK cells were enriched through density gradient separation using RosetteSep NK cell Enrichment Cocktail according to the manufacturer’s protocol (StemCell Technologies), followed by B-cell depletion using anti-CD19 Dynabeads (ThermoFisher Scientific). The resulting NK cell population was > 90% CD56^+^ and CD3^−^. The following tumor cell lines obtained from ATCC were used: NK-92 (NK cell line), HCT116 (colorectal carcinoma), HCT-15 (Dukes type C colorectal adenocarcinoma), DU145 (prostate carcinoma), PC3 (prostate adenocarcinoma), SK-BR-3 (breast adenocarcinoma), T-4D7 (mammary gland ductal carcinoma), OVCAR-3 (ovarian adenocarcinoma), WM9 (metastatic melanoma), and U87 (glioblastoma). The NK cell line KHYG-1 was obtained from the German Collection of Microorganisms and Cell Cultures (DSMZ). All cell lines were cultured in complete RPMI-1640 medium (cRPMI), supplemented with 10% FBS, 1% penicillin/streptomycin, 1% sodium pyruvate, and 50 mM 2-mercaptoethanol (NK-92 in cRPMI with 20% FBS). NK-92 and KHYG-1 cell cultures were supplemented with 500 IU/ml human recombinant IL-2.

### Flow cytometry

NK cell purity and viability was tested by anti-human CD56-AF647 (B159, BD Biosciences), CD3-AF700 (OKT3, BioLegend), CD19-PerCep-Cy5.5 (SJ25C1, BioLegend), CD14-FITC (M5E2, BioLegend), and Fixable Viability Dye eFluor780 (ThermoFisher Scientific). Cancer cells were stained with PVR-PE (SKII4, BioLegend), Nectin2-PE-Cy7 (TX31, BioLegend), B7H6-AF647 (FAB7144A, R&D Systems), or MICA/B-FITC (mAb, a gift from A. Steinle, Frankfurt, Germany). Cancer cell death was measured by cellular uptake of propidium iodide (PI), upon co-culture of 100 µl cells (5 × 10^5^/ml) with 20 µg purified EVs for 24 h. Medium alone or cancer cell-derived EVs served as negative controls. Cells were analyzed by flow cytometry (BD Fortessa, BD Biosciences) and FlowJo software.

### EV production and isolation

Freshly isolated NK cells (2 × 10^6^/ml), NK-92 cells (5 × 10^5^/ml), or KHYG-1 cells (5 × 10^5^/ml) were cultured in 10 ng/ml human recombinant IL-15 alone, or in combination with 10 ng/ml human recombinant IL-12 and IL-18 (all from R&D Systems) in serum-free AIM-V medium for 48 h. Additionally, NK cells were stimulated with anti-CD16-coated pan-mouse Dynabeads (ThermoFisher Scientific) for 48 h in AIM-V medium. Culture supernatants were spun at 2.000* g* for 30 min, and filtered (0.22-µm pore-size). EVs were precipitated using Total Exosome Isolation Reagent (ThermoFisher Scientific) according to manufacturer’s protocol, resuspended in PBS, and stored at − 20 °C. Alternatively, EVs were resuspended in 1 ml PBS and further purified through Size-exclusion chromatography (SEC) using 10 ml Sepharose 4B (GE Healthcare) columns into ten fractions á 1 ml with PBS as elution buffer, and concentrated with Amicon Ultra 0.5 mL centrifugal filters (Millipore). Protein concentration was measured with Pierce BCA Protein Assay Kit (ThermoFisher).

### Nanoparticle tracking analysis (NTA)

EVs diluted in PBS (1:100) were analyzed using an LM10 nanoparticle tracking analyzer with a 532-laser (Malvern Panalytics). Samples were analyzed under constant flow conditions (flow rate = 20) at 25 °C, and 3–10 × 60 s videos were captured. Data were analyzed using NTA software with a detection threshold of 5 and bin size 2.

### Transmission electron microscopy

EVs were imaged by negative stain electron microscopy. A 400 mesh copper grid with carbon-coated formvar film were placed on 20 µl EVs and incubated for 10 min. The grid was washed twice in water, excess liquid removed, and transferred to a drop of 2% uranyl acetate for 30 s, dried and subjected to microscopy with a Tecnai G^2^ Spirit TEM (FEI, The Netherlands) equipped with a Morada digital camera and RADIUS imagining software.

### Western blotting

Purified EVs (20 µg) were lysed in 2 × Triton X-100 lysis buffer (50 mM Tris/300 mM NaCl pH 7.6%, 2% protease inhibitor cocktail (Roche), 2% Triton X-100) for 10 min on ice, spun at 10.000 g for 10 min, and mixed with non-reducing or reducing SDS sample buffer and boiled for 5 min. Samples were run on 10 or 12% SDS-PAGE Criterion gels (Bio-Rad), proteins transferred onto PVDF membranes (ThermoFisher Scientific), and blocked with 5% dry milk. Membranes were incubated overnight at 4 °C with the primary antibodies CD63 (Ts63), CD81 (M38), and TSG101 (4A10) from ThermoFisher Scientific, Perforin (#1001103) and Granzyme B (#2103A) from R&D Systems, and FasL (M143, Santa Cruz Biotechnology). Blots were probed with goat anti-rabbit IgG-HRP or goat anti-mouse IgG-HRP (Bio-Rad), and developed by Pierce ECL Western Blotting Substrate (ThermoFisher Scientific).

### Spheroid apoptosis

Spheroids were generated by seeding 1–3000 tumor cells into a Nunclon Sphera round-bottom 96-well plate (ThermoFisher Scientific) in 200 µl cRPMI. On day 3, medium was renewed, and 20 µg of EVs were added to appropriate wells in duplicates, together with 12 µM CellEvent Caspase-3/7 Green Detection Reagent (ThermoFisher Scientific). Spheroids were monitored every hr in an IncuCyte S3 instrument (Sartorius) for up to 5 days. Spheroid apoptosis was also monitored by fluorescence microscopy (FLoid Cell Imaging Station), and further analyzed by ImageJ. To assess involvement of receptor-ligand interactions, EVs and spheroids were co-cultured in presence of 10 µg/ml of antibodies toward DNAM-1 (DX11, Invitrogen), NKp46 (9E2, BioLegend), NKp30 (P-30–15, BioLegend), FasL (NOK-1, Invitrogen), NKG2D (5C6, eBioScience), or MICA/B.

### NK cell cytotoxic assay

A flow cytometry-based protocol to simultaneously assess cytolysis and degranulation by NK cells was adapted from Oei et al. [32]. Primary NK cells, NK-92 or KHYG-1 cells were mixed with CFDA-SE-stained target cells at 10:1 or 1:1 effector:target ratio in 200 µl cRPMI in 96-well round bottom plates, and incubated 2 h at 37 °C in presence of a CD107a-BV510 antibody (H4A3, BioLegend). After incubation, cells were stained with CD56-AF647, CD3-AF700 and Fixable Viability Dye eFluor780. Tumor targets were gated as CFSE^+^ cells, and dead cells defined as CFSE^+^eFluor780^+^ events. NK cells were gated as CFSE^−^CD56^+^CD3^−^ cells, and percentage degranulating CD107a^+^ cells calculated from this gate.

### Histology

EVs (20 µg) were stained with 10 µM CMTMR (ThermoFisher Scientific), and washed using exosome spin columns (MW3000, ThermoFisher Scientific). HCT116 spheroids were incubated with CMTMR-stained EVs and 12 µM of CellEvent Caspase-3/7 Green Detection Reagent for 3 days. Single tumor spheroids were fixed with 4% formaldehyde for 1 h, aligned on the bottom of a plastic mold and snap-frozen in OCT compound (Tissue-Tek) using liquid nitrogen. Cryosections of 10 µm, with 20 µm intervals, were obtained using a Leica cm3050s cryostat. Sections were stained with DAPI and embedded in Prolong Gold antifade mounting medium (ThermoFisher) and analyzed using Olympus fluorescence microscope.

### Proteomic analysis

Triplicate samples of EVs in PBS were subjected to LC–MS/MS analysis. EVs were lysed in SDS and proteins precipitated using MagReSyn Amine beads (20 mg/ml, ReSyn BioScience) in 70% acetonitrile. Beads were washed in acetonitrile and 70% ethanol, and proteins reduced in 10 mM DTT in 50 mM ammonium bicarbonate for 30 min at 37 °C, and alkylated by iodoacetamine (20 mM). Proteins were digested with 1 µg trypsin at 37 °C overnight, and peptides captured, concentrated, and desalted using Evotip C18. Samples were analyzed by an EvosepOneLC coupled to a quadrupole–Orbitrap (QExactive HF) mass spectrometer (ThermoElectron) using a 15 cm C18 column with setting 30 samples/day. The mass spectrometer was operated in data-dependent mode to automatically switch between MS and MS/MS acquisition. MS raw files were submitted to MaxQuant software version 1.6.17.0 for protein identification [33]. Minimal unique peptides were set to 1, and FDR allowed was 0.01 for identification. The Swissprot human database was used with a requirement of minimum 2 valid values in at least one group. Data visualization was done using FunRich version 3.1.4 [34] and GraphPad. The mass spectrometry proteomics data have been deposited to the ProteomeXchange Consortium via the PRIDE partner repository (identifier PXD027284).

### Statistical analysis

All data are expressed as mean ± SEM of at least three independent experiments using GraphPad Prism. Statistical analysis were performed using the unpaired Mann–Whitney *U*-test.

## Results

### Resting and cytokine-activated NK cells yield similar output of EVs

The level of EV production, EV size, and expression of EV markers was compared in EVs prepared from primary NK cells, NK-92, or KHYG-1 cells cultured in either IL-15 alone or in combination with IL-12 and IL-18 for 48 h. For primary NK cells, stimulation through the activating receptor CD16 was included as an alternative activation pathway. This receptor is not expressed by the NK cell lines. EVs were precipitated from cell culture supernatants, and resuspended in a volume reflecting the cell numbers of the cultures.

**Fig. 1:**
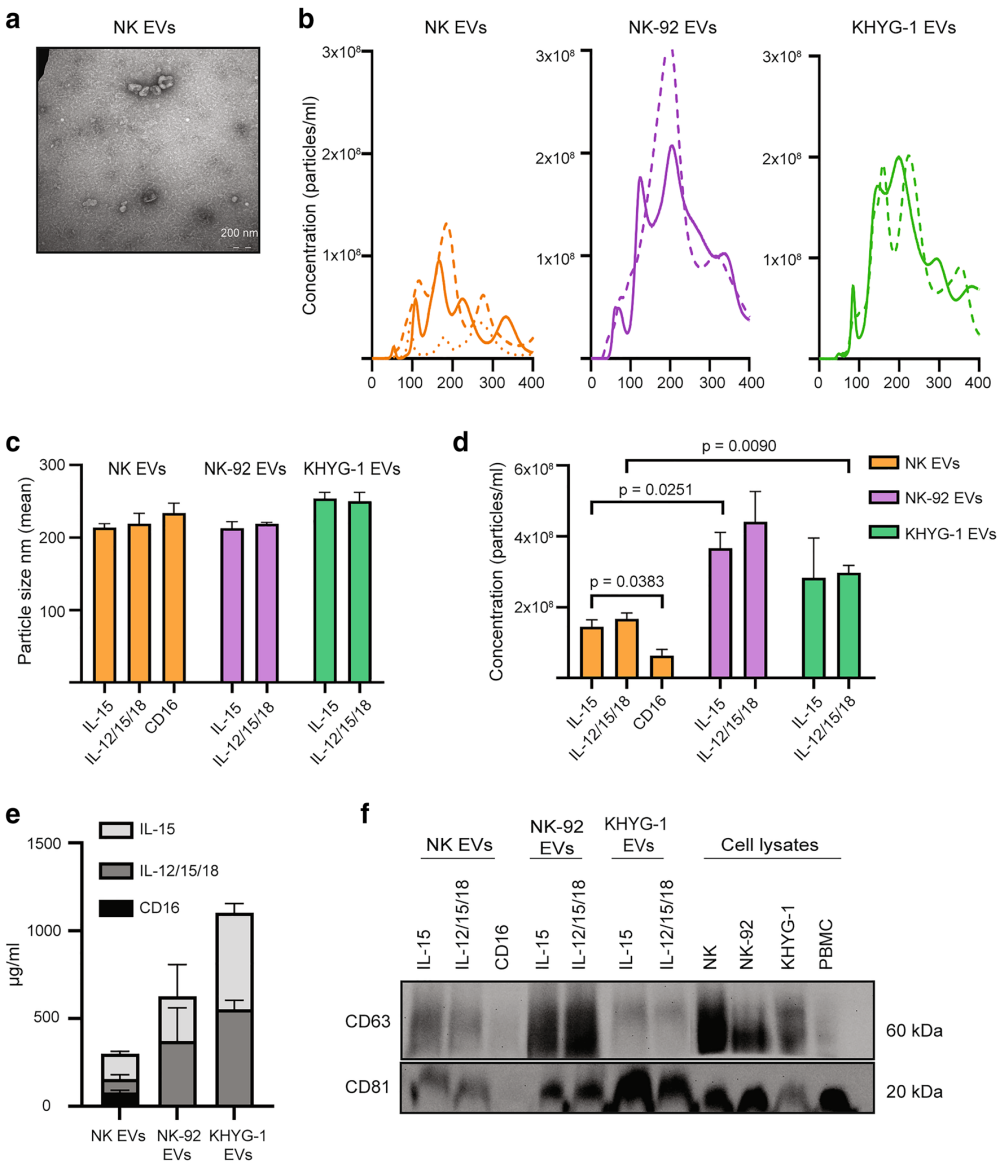
Comparison of EVs derived from IL-15 or IL-12/15/18-stimulated NK cells, NK-92 or KHYG-1 cells. **a** TEM image of EVs derived from IL-12/15/18-stimulated NK cells. Scale bar 200 nm. **b** NTA analysis of EVs derived from indicated stimulations of NK cells, NK-92 cells or KHYG-1 cells. Representative of one of three separate experiments. Particle size **(c)** and particle concentration **(d)** as measured by NTA of EVs derived from indicated stimulations of NK cells, NK-92 cells or KHYG-1 cells. Data are presented as the mean ± SEM of three separate experiments. **e** BCA analysis of EVs derived from indicated stimulations of NK cells, NK-92 cells or KHYG-1 cells. Data are presented as the mean ± SEM of five separate experiments. **f** Western blot analysis using 20 µg of indicated EV isolates and corresponding cellular lysates. Representative of three independent experiments

Electron microscopy images of EVs from NK cells confirmed successful isolation of vesicles of cup-shaped morphology in the size range 60–125 nm (Fig. 1a). Particle concentration measurements by NTA showed EV concentration from the two NK-cell lines compared to NK cells (Fig. 1b), but a similar EV-concentration from IL-15 or IL-12/15/18 stimulated cells for all three cell types (Fig. 1b). CD16-stimulated NK cells generated lower amounts of EVs compared to cytokine-treated cells (Fig. 1d). EV size was comparable between the different stimuli conditions and cell types (Fig. 1c). Protein concentration measurements largely mirrored the NTA measurements (Fig. 1e). Finally, the different EV isolates expressed the EV markers CD63 and CD81 (Fig. 1f). Notably, KHYG-1 EVs had strong expression of CD81 relative to CD63, whereas CD81 and CD63 signal ratios were comparable for NK- and NK-92 EVs. Expression of both markers were low in EVs from CD16-stimulated NK cells. In summary, although the IL-12/15/18 cytokines yield NK cells with enhanced effector functions, this is not reflected in EV output.

### EVs from IL-12/15/18-stimulated cells have enhanced ability to kill tumor spheroids.

**Fig. 2:**
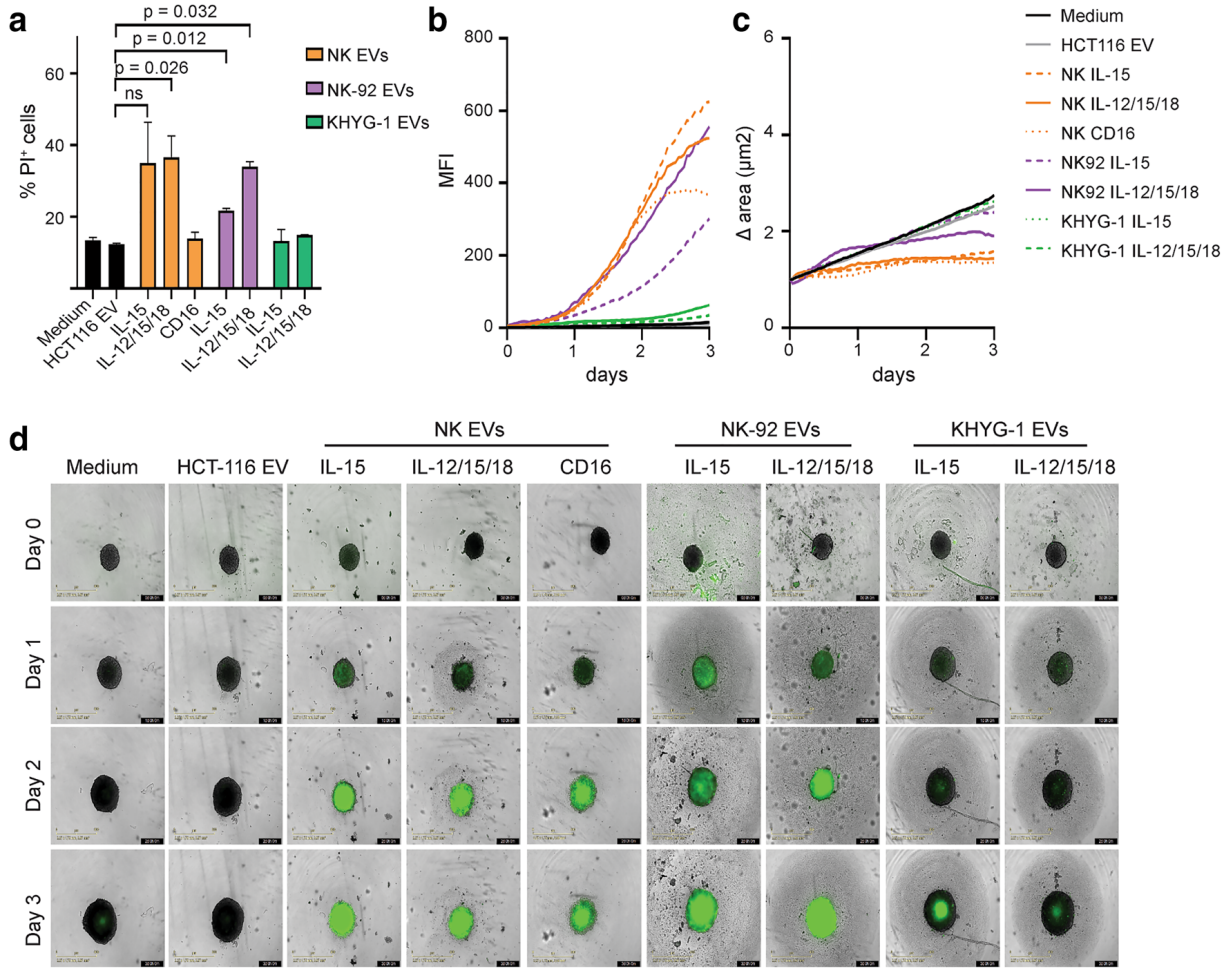
EVs derived from primary NK cells or NK-92 cells induce apoptosis of HCT116 colon cancer cells. **a** Frequencies of propidium iodide positive cells upon treatment of HCT116 cell cultures with indicated EV isolates (20 µg) for 24 h. **b-c** Induction of apoptosis of HCT116 spheroids upon treatment with indicated EV isolates for up to 3 days. Apoptosis was detected with Caspase 3/7 green detection reagent, and green fluorescence **(b)** and spheroid size **(c)** was monitored every hr using the Incucyte S3 platform. Data are representative of one of five experiments. **d** Representative images taken by the S3 Incucyte at time of EV application (day 0), and days 1–3 after EV application

We next compared the killing potential of the different EV isolates against the colon cancer cell line HCT116. EVs from IL-15- or IL-12/15/18-stimulated NK cells or NK-92 cells induced all cell death, and EVs from activated NK-92 cells induced more death than EVs from IL-15-stimulated cells (Fig. 2a). KHYG-1 EVs induced surprisingly little cell death. To better mimic a solid tumor, we generated HCT116 spheroids and assessed induction of apoptosis and spheroid growth over 3 days (Fig. 2b-d). Both NK-EVs and NK-92-EVs induced an increased caspase 3/7 activity in the spheroids, with slightly higher apoptosis from IL-12/15/18-genereated EVs. Again, KHYG-1 EVs did not induce apoptosis over background.

**Fig. 3:**
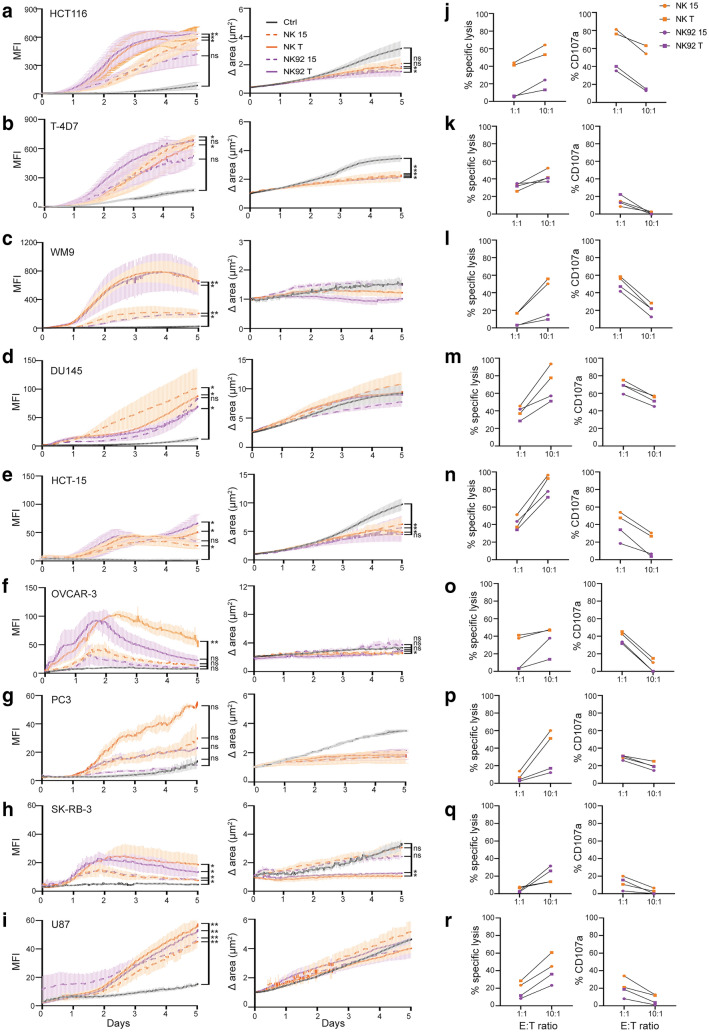
EVs from primary NK cells and NK-92 cells broadly target tumor spheroids. **a-i** Measurement of apoptosis of indicated tumor spheroids with EVs from IL-15 or IL-12/15/18-stimulated primary NK cells or NK-92 cells measured as increase in the MFI signal of Caspase 3/7 green detection reagent (left panels) or spheroid size (right panels) using the Incucyte S3 platform. Spheroids were monitored every hr for 5 days. **j-r** Cytotoxic killing assay (left panels) or degranulation assay (right panels) of donor NK cells or NK-92 cells stimulated for 48 h with IL-15 or IL-12/15/18 toward indicated cancer cells

We next compared the apoptotic effect of EVs from IL-15 or IL-12/15/18-stimulated NK cells or NK-92 against a panel of nine tumor spheroids over 5 days (Fig. 3 and Suppl. Figure 1). HCT116, T-4D7, and WM9 were most sensitive to EVs, both from NK cells and NK-92 cells (Fig. 3a-c), followed by DU145, HCT-15, and OVCAR-3 (Fig. 3d-f). PC3, SK-BR-3, and U87 were the least sensitive in terms of measured apoptotic signal (Fig. 3g-i). IL-12/15/18-generated EVs, from both NK cells and NK-92 cells, induced significantly higher apoptotic signals by day 5 than negative controls. Apoptosis induced by IL-12/15/18-generated EVs were for some spheroids in the same range by day 5 as that induced by IL-15-generated EVs (HCT-15, HCT116, DU145, T-4D7, U87), or significantly higher (WM9, OVCAR-3, SK-BR-3). The kinetics of the apoptotic signal varied for the different tumor spheroids, and for OVCAR-3 the apoptotic signal induced by IL12/15/18- generated EV was higher at day 2 compared to IL-15-generated EVs (*p* = 0.02 and *p* = 0.003, respectively). Blunted growth was only observed for T-4D7, SK-BR-3 (only with IL12/15/18-derived NK-EVs), and HCT116 spheroids.

For comparison against EVs, the donor NK cells and NK-92 cells were tested in killing assays (Fig. 3j-r). They showed highest killing capacity of HCT-15 and DU145 cells. PC3 and SK-BR-3 were relatively resistant to both cellular cytotoxicity and EV-induced apoptosis. Interestingly, although EVs induced high levels of apoptosis of HCT116, T-4D7 and WM9, this was not reflected in cellular cytotoxicity, which was particularly low toward WM9 (Fig. 3r). This suggest that EVs may target cancer cells not killed by the cellular counterpart.

### EVs contain cytolytic proteins dictated by their cell of origin

**Fig. 4:**
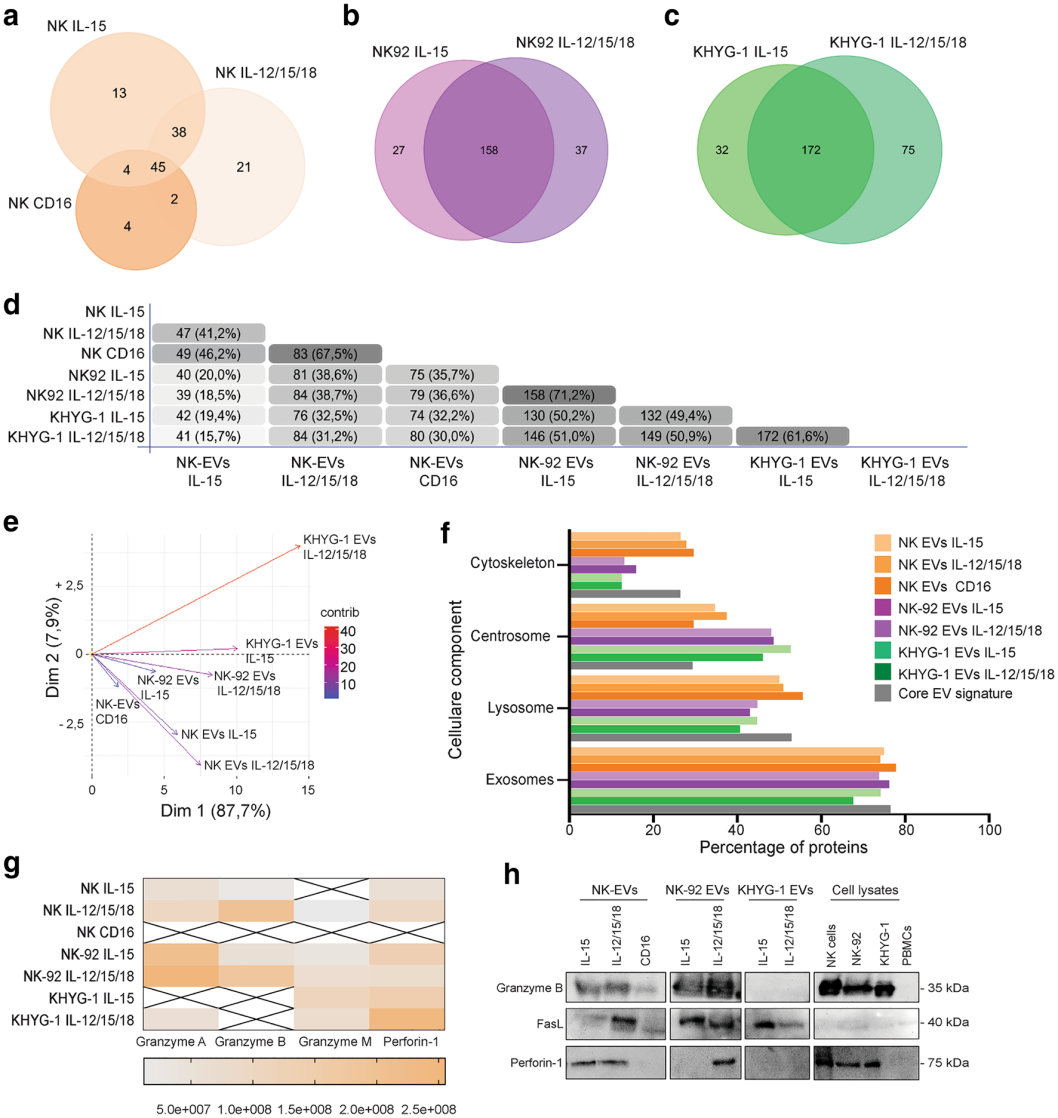
Proteomic comparison of EVs derived from NK cells, NK-92 and KHYG-1 cultured in presence of IL-15 or IL-12/15/18. Venn diagrams depicting number of identified proteins in at least two of three replicates of EVs from **(a)** primary NK cells, **(b)**, NK-92 cells or **(c)** KHYG-1 cells stimulated with IL-15 or IL-12/15/18 for 48 h, and CD16 for 48 h for NK cells. **d** Comparative Venn diagram depicting percent similarities in the protein profile of the 7 different EV isolates. **e** PCA analysis of the protein profile of the seven EV isolates based on the median intensity of proteins. The variability explained by the first and second components of the PCA is indicated by arrow direction and length. **f** Cellular component analysis of proteins identified in EVs from NK cells, NK-92 or KHYG-1 by FunRich analysis showing percentage of identified proteins within the depicted pathways. Enrichment within depicted pathways were significant for all EV isolates (*p* < 0.001). **g** Heatmap showing the median protein intensity values of cytolytic proteins detected in the EV isolates. **h** Western blot analysis of 20 µg EVs derived from NK cells, NK-92 or KHYG-1 cells stimulated by the indicated cytokines or CD16. Whole cell lysates of indicated cells as controls. The data is representative of three independent experiments

The observed differences in tumor-targeting abilities prompted a comparative protein profiling. Triplicate samples were subjected to LC–MS/MS, and a total 534 proteins were identified across all samples and replicates after subtraction of contaminants. Further analyses were restricted to proteins detected in at least two of the three replicates (Suppl. Table 1). 127 proteins were identified in EVs from primary NK cells, 45 of which were shared by EVs derived from the three different stimulations (Fig. 4a). 222 and 279 proteins were identified in NK-92 EVs or KHYG-1 EVs, respectively (Fig. 4b-c). The similarity of the proteome recovered from EVs isolated from either IL-15- or IL-12/15/18-stimulated cells ranged from 61.6 to 71.2% (Fig. 4d). PCA analysis showed that EVs tended to cluster based on their cell of origin (Fig. 4e). Cellular component analysis showed high enrichment of exosomal proteins for all samples (*p* < 0.001)(Fig. 4f). A core of 34 proteins were identified in all seven EV isolates, and showed a similar pathway enrichment as individual EV isolates (Fig. 4f). Cytolytic proteins were differentially distributed. While FasL was detectable in EVs from NK cells, NK-92 and KHYG-1 cells, granzyme B and perforin were only detected in EVs from NK cells and NK-92 cells (Fig. 4G and H). Granzyme B was similarly expressed in EVs from IL-15 vs IL-12/15/18-stimulated cells. Both granzyme B and perforin were present at low levels in EVs from CD16-stimulated cells. Taken together, the data show that the EV protein profile varies between NK- cell sources, and may also explain why KHYG-1 EVs appear less potent.

### Granzyme B and perforin are concentrated in purified vesicles

**Fig. 5:**
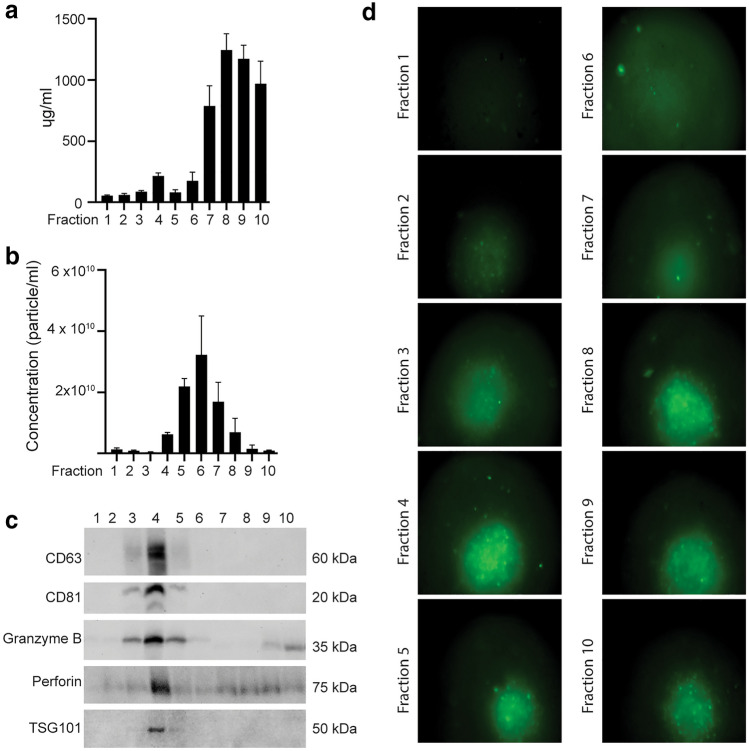
Granzyme B and perforin are expressed in vesicular fractions bearing CD81 and CD63. **a** Protein concentration by BCA analysis of SEC fractions 1–10 (1 ml fractions) of an NK-92 EV precipitate. Data are presented as the mean ± SEM of four independent experiments. **b** Particle concentration measured by NTA analysis of SEC fractions 1–10 of NK-92 EV precipitate. Data are presented as the mean ± SEM of four independent experiments. **c** Measurement of caspase 3/7 activation by green fluorescence of 3 day-old HCT116 spheroids cultured in presence of 20 µl of indicated fractions for 24 h, and imaging by FLoid cell imaging station

Isolation of EVs by precipitation may result in co-precipitation of protein aggregates. To verify that the observed tumor cell apoptosis was induced by EVs, the EV precipitate was further purified by SEC. Ten fractions were analyzed with the highest protein concentration contained in fractions 7–10, and the highest particle concentrations were measured in fractions 5–7 (Fig. 5a,b). Western blot analysis showed that both perforin and granzyme B co-localized in fraction 4 together with the small EV markers CD81 and CD63, indicating that small EVs indeed contain cytolytic proteins. Low levels of granzyme B and perforin were also detected in the later fractions as putative soluble proteins (Fig. 5c). Finally, we demonstrate that the contents of fraction 4 induced apoptosis of HCT116 tumor spheroids (Fig. 5d).

### EVs enter the spheroid core, and apoptosis is partly dependent on NKG2D.

**Fig. 6:**
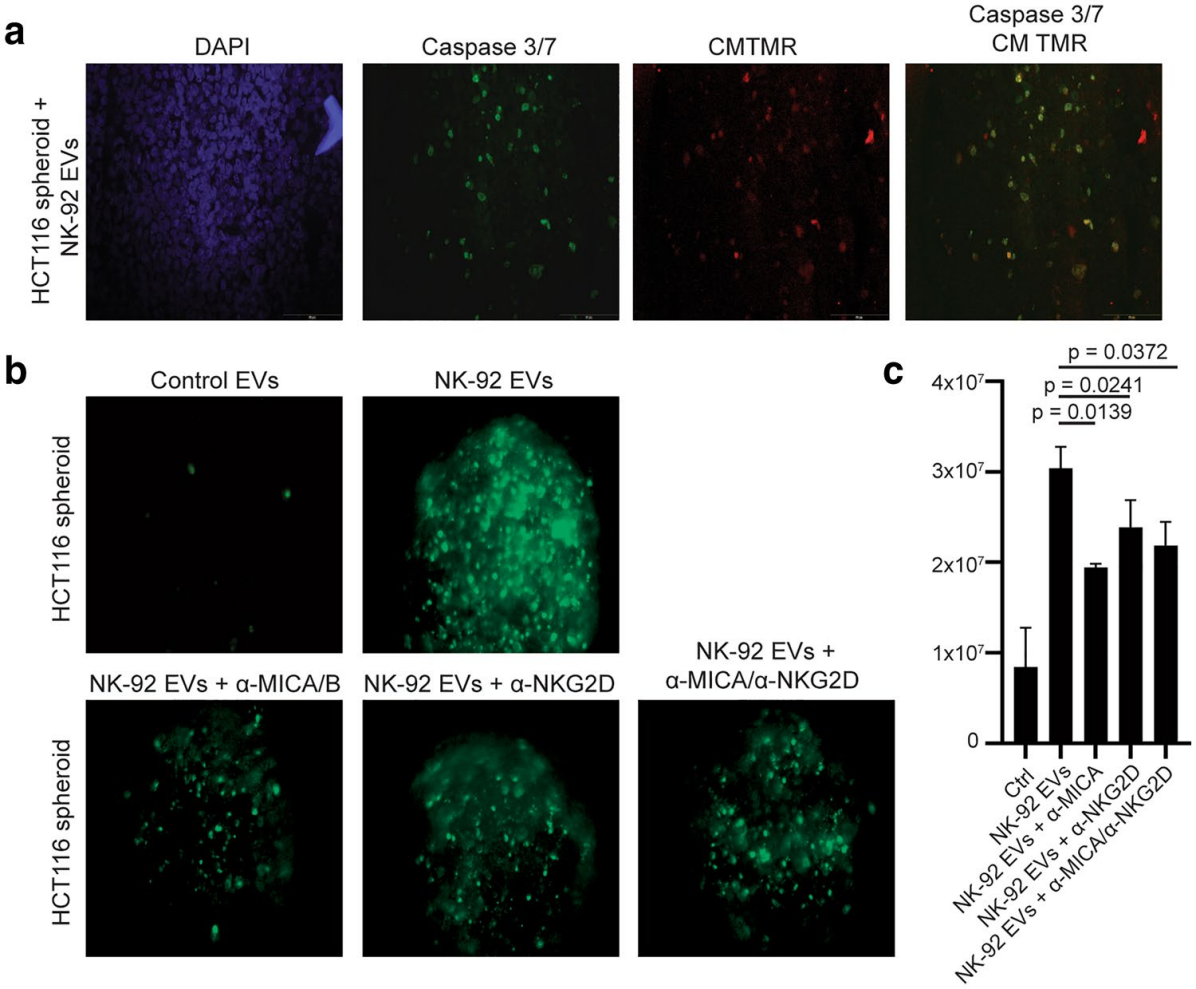
EVs derived from NK cells enter the spheroid core, and spheroid apoptosis is dependent on NKG2D–MICA/B interaction. **a** HCT116 spheroids were incubated with CMTMR-stained EVs (red) derived from NK cells cultured in IL-12/15/18 for 24 h in presence of caspase 3/7 green detection reagent. The spheroids were fixed, sectioned, stained with the nuclear dye DAPI (blue), and analyzed by fluorescence microscopy. The images are representative of five different spheroids. **b-c** HCT116 spheroids were cultured with EVs derived from NK-92 cells stimulated with IL-12/15/18 in the presence of blocking antibodies toward NKG2D, MICA/B, or a combination of both. The images **(b)** is representative of four independent experiments, and the graph **(c)** shows the median SEM of four independent experiments

We next tested the ability of EVs to penetrate the spheroids. EVs derived from IL-12/15/18-stimulated NK cells were labelled with the red dye CMTMR to allow their identification. Spheroids were fixed and sectioned after 3 days culture with EVs. Figure 6a shows that EVs are detected within the tumor spheroids and co-localizing with apoptotic cells, indicating ability to enter the spheroid. We finally addressed the mechanism by which the EVs interact with the tumor cells, and hypothesized the involvement of activating NK cell receptors or death receptors. The tumor cell lines all variably express the DNAM-1 ligands CD112 and CD155 (Suppl Fig. 2), low levels B7-H6 (NKp30 ligand), while the NKG2D ligands MICA/B were expressed by HCT-15, HCT116, OVCAR-3, T-4D7, WM9, and DU145. HCT116 spheroids co-incubated with NK-92 EVs in presence of antibodies toward either MICA/B, NKG2D, or both, showed a decrease in apoptotic signal (Fig. 6b, c). Incubations with antibodies toward DNAM-1, NKp30, NKp46, or FasL had no effect (data not shown). These data suggest that specific receptor-ligand interactions are involved in NK-cell derived EV-mediated tumor cell apoptosis.

## Discussion

In this study, we have performed a comparative characterization of EVs derived from either primary NK cells or the NK cell lines NK-92 and KHYG-1 cultured under either resting or activating conditions. The study is thus the first to directly compare EVs from primary NK cells versus NK cell lines.

Short-term cultures of NK cells with the combination of cytokines IL-12, IL-15, and IL-18 yields NK cells with enhanced ability to eradicate tumors in vivo [5–7]. We hypothesized that NK cells cultured under these conditions yield a higher EV output, but we show in this study that there is comparable release of EVs from resting versus activated NK cells. Previous data are in line with our data, in that comparable amounts of exosomes were found when comparing resting versus IL-2-activated primary human NK cells [25, 35], although these studies were based on protein quantifications alone. We also subjected NK cells to activation through the Fc receptor CD16 that mediate antibody-dependent cellular cytotoxicity. Stimulation through CD16 yields a strong activation signal in NK cells, but generated only low levels of EVs as judged by NTA and testing for CD63 and CD81 expression. This could indicate that EV release in NK cells is predominantly induced via cytokines.

Previous studies of NK-cell derived EVs were based on separation via ultracentrifugation or precipitation. We isolated EVs via precipitation, although the EV isolate will not be as pure as that obtained via ultracentrifugation. A major concern was the possible co-precipitation of protein aggregates containing perforin and/or granzymes that could be mediating the observed tumor cell apoptosis. Further purification of the EV precipitate by SEC demonstrated the co-localization of the small EV markers CD81 and CD63 with perforin and granzyme B, demonstrating that cytolytic proteins are enriched within the EV fraction and not merely present as soluble contaminants. Importantly, apoptosis was induced by the purified EV fraction, indicating that tumor cell death is indeed mediated by the EVs.

Interestingly, a recent study demonstrated that NK cells secrete cytolytic proteins in the form of particles surrounded by a coat of glycoproteins [36]. We cannot exclude that such particles may be present in our EV precipitates, but we detected low levels of perforin and granzymes in EV isolates from CD16-stimulated NK cells, which should induce release of cytolytic granule contents. Cytolytic granules in cytotoxic T cells are shown to contain intraluminal vesicles that may be secreted [37], but T cells also release small microvesicles upon contact with target cells [38]. We hypothesize that the EVs secreted from CD16-stimulated NK cells could represent microvesicles, in which case these would not incorporate cytolytic proteins.

KHYG-1 EVs were functionally different from EVs derived from primary NK cells or NK-92 cells in that they did not induce tumor cell apoptosis. We observed that KHYG-1 EVs contained high levels of CD81 relative to CD63, a skewing that was not apparent for EVs from NK cells or NK-92. Western blots indicated no skewing in expression of CD81 versus CD63 in KHYG-1 cell lysates. Interestingly, a recent study suggested that small EVs expressing mainly CD81 represent vesicles budding off from the plasma membrane [39] [40]. This could explain the absence of granzyme B within KHYG-1 EVs, which would be sorted to intraluminal vesicles en route to cytolytic granules.

We observed a tendency for higher levels of spheroid apoptosis of IL-12/15/18-generated EVs compared to EVs derived from cells cultured in IL-15 alone, which would argue for the use of EVs derived from cytokine-activated NK cells for therapeutic use. This difference was pronounced for WM9, OVCAR-3 and SK-RB-3 spheroids. The explanation for this functional difference is currently unknown, and the MS analysis did not reveal major differences between EVs from IL-15 vs IL-12/15/18-stimulated cells that could explain differential function, but we speculate there may be differences in surface markers that were not picked up by the MS analysis. Interestingly, the WM9 and SK-RB-3 cell lines represent cell lines not efficiently killed by NK cells, indicating that NK-cell derived EVs may target cells independently of their donor cell.

Mechanistically, we pinpoint NKG2D as a receptor mediating the interaction between NK-cell derived EVs with HCT116 spheroids. The HCT116 cells expressed high levels of the NKG2D ligands MICA/B, as did the cell lines that were targetable by NK-EVs. Interestingly, we show that the three least targetable tumor cell lines, U87, PC3, and SK-RB-3 expressed very little MICA/B. In support of our data, exosomes from NK cells were previously shown to contain relatively high levels of NKG2D [24, 26]. The tumor cell lines expressed ligands for the DNAM-1 receptors, but we did not observe any reduction of apoptosis of HCT116 spheroids when attempting to block DNAM-1. This was also the case for FasL, although FasL was expressed by all EV isolates. In contrast, a recent study showed that blockade of DNAM-1 ligands led to a slight reduction in killing potential of EVs derived from primary NK cells against by the lymphoblastic cell line NALM-18 [25]. The same study suggested that the EVs were internalized, and that there was a correlation between amount of internalized EVs and the cytotoxic effect on the tumor cells [25]. Our own study indicates that EVs are able to infiltrate the spheroid structure, as we were able to detect EVs within cells in the HCT116 spheroid core.

In summary, we show that EV output is similar under resting or cytokine-activating conditions, and that EVs derived from either primary NK cells or the NK-92 cell lines show similar ability to induce apoptosis of tumor spheroids, with a slight enhanced function of EVs derived IL-12/15/18-stimulated cells. We thus conclude that an optimal NK-cell derived EV therapeutic product could be derived from NK-92 cells stimulated with IL-12/15/18 cytokines. Importantly, future studies should address methodology to separate subsets of EVs with cytolytic function, in contrast to bulk EVs obtained by precipitation, SEC, and even ultracentrifugation. Bulk EVs are heterogeneous in terms of size, cargo, and function, and also derive from different sources within the cell or from the plasma membrane. In the light of our findings, subsets of cytolytic NK-EVs may represent an ideal candidate for future therapeutic applications.

### Supplementary Information

Below is the link to the electronic supplementary material.

Supplementary file1 (TIF 1096 kb)

Supplementary file2 (TIF 3608 kb)

## Data Availability

Proteomics data are available through PRIDE (accession number PXD027284).

## List of abbreviation

EV: Extracellular vesicles
