# Supplementary figures and images for: Comparison of characteristics and tumor targeting properties of extracellular vesicles derived from primary NK cells or NK-cell lines stimulated with IL-15 or IL-12/15/18
Source: Cancer Immunol Immunother. 2022 Feb 4;71(9):2227–38. doi: 10.1007/s00262-022-03161-0 (PMC9374793; doi:10.1007/s00262-022-03161-0)

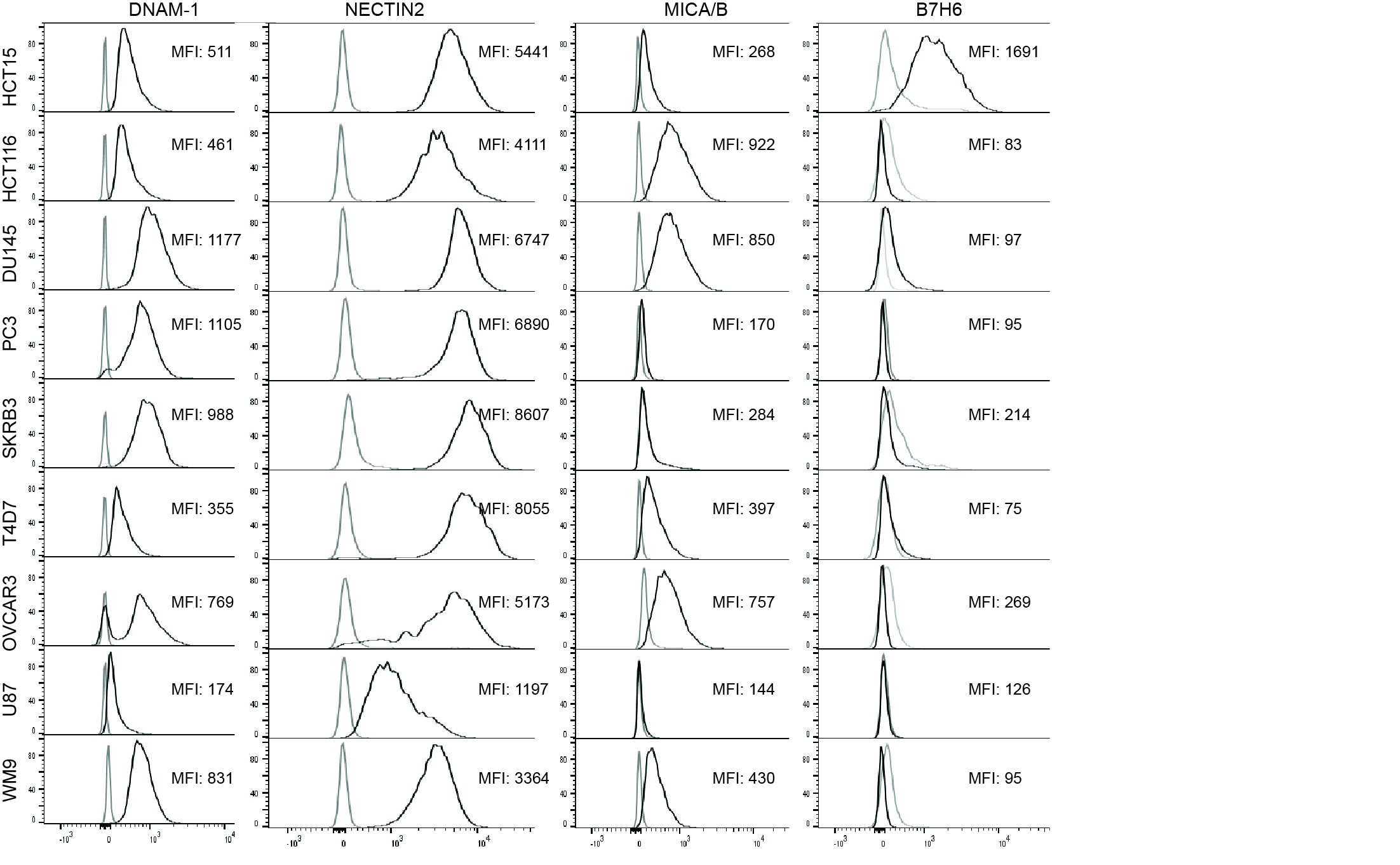

Supplement: Supplementary file 1 — Supplementary file1 (TIF 1096 kb) [file 262_2022_3161_MOESM1_ESM.tif]

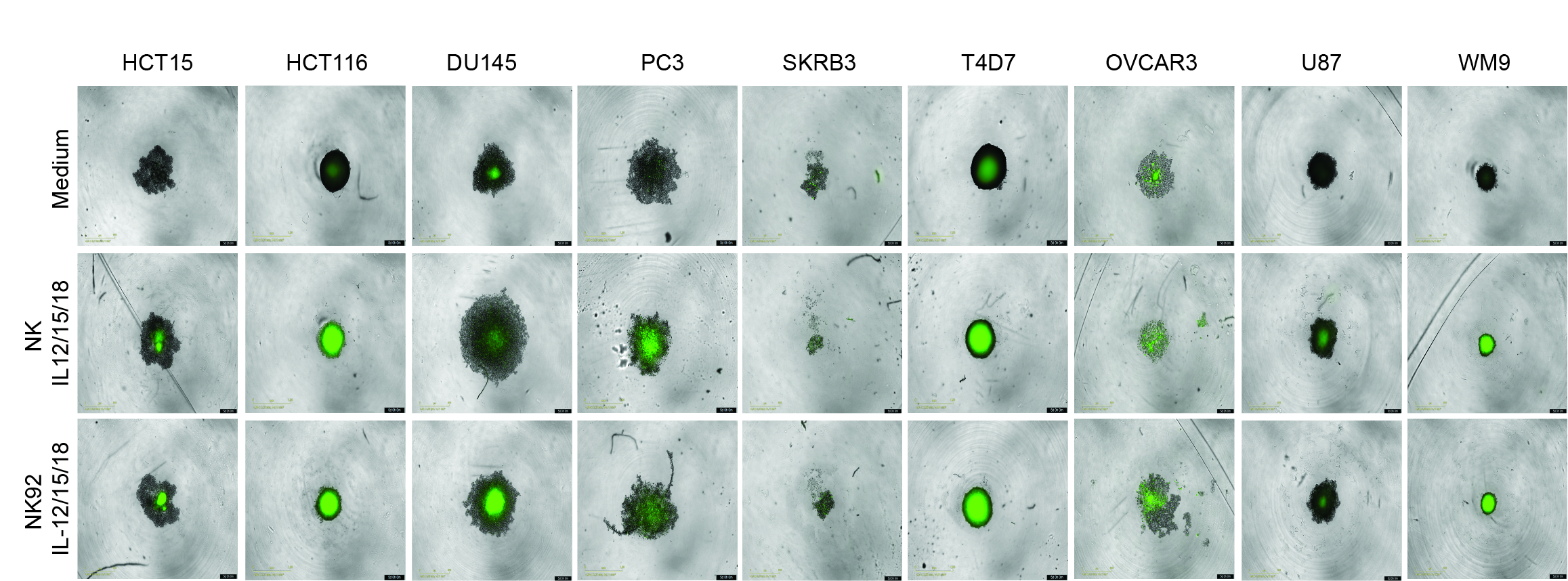

Supplement: Supplementary file 2 — Supplementary file2 (TIF 3608 kb) [file 262_2022_3161_MOESM2_ESM.tif]
